# Nomophobia and its predictors in undergraduate students of Lahore, Pakistan

**DOI:** 10.1016/j.heliyon.2020.e04837

**Published:** 2020-09-06

**Authors:** Elizabeth Schwaiger, Rameen Tahir

**Affiliations:** a Department of Psychology, Forman Christian College (A Chartered University), Lahore, Pakistan; b Forman Christian College (A Chartered University), Lahore, Pakistan

**Keywords:** Psychology, Smartphones, Nomophobia, Undergraduate students, Pakistan

## Abstract

Increasing rates of smartphone use in Pakistani undergraduate students, coupled with a dearth of research, indicate a need for a better understanding of the effects of Smartphone use on this population. This cross-sectional study therefore aimed to measure rates of nomophobia and its predictors among this understudied population. A total of 138 undergraduate students of a private university in Lahore, Pakistan completed a demographics questionnaire and the Nomophobia Questionnaire (NMP-Q) as part of a larger study. All students reported some level of nomophobia, with the largest proportion reporting moderate levels. Gender differences were seen in NMP-Q scores, with women reporting greater levels of nomophobia, specifically within the “Not being able to communicate” subscale of the NMP-Q. Multiple linear regression indicated that, while hours of usage per day, number of times per day checking phone, and amount of time in university were all correlated with NMP-Q scores, however, the only significant predictor was hours of usage per day [R = .331, R^2^ = .109, F (3, 116) = 4.748, p < .05]. The results of this study indicate that, as use of smartphones continues to increase in this developing nation, monitoring nomophobia and its correlates is of great importance, and carries with it implications at the societal and university policy levels.

## 1 Introduction

Pakistan is one of the leading nations in statistically significant increases in Smartphone ownership. With a 7% increase in Smartphone usage between 2013 (8%) and 2015 (15%), it ranks 11^th^ in the list of emerging countries who have seen significant changes in Smartphone ownership over that period [1]. Moreover, the majority of those polled use their Smartphones to access the Internet daily, with the greatest proportion of those using the Internet to access social media sites [1]. Given the sharp increase in Smartphone ownership in Pakistan - especially among millennials and those with higher education and access to higher income [1] - it becomes important to also understand the corresponding level of nomophobia, or fear of being without one's phone [2, 3].

Despite increasing levels of smartphone use, however, only one study has been conducted, comparing levels of nomophobia in Pakistani students to those in Turkey. This study found that Pakistani students in general had lower levels of nomophobia than their counterparts in Turkey and that there were no gender differences in levels of nomophobia in Pakistani students [4]. Research in the neighboring country of India, a country which shares some cultural context with Pakistan, provides wide estimates of rates of nomophobia, ranging from 6% in nursing students [5] to 72% in medical students [6] and between 40% [7] to almost 100% in Indian college students [8, 9]. Some studies indicate that, even for those who are not currently experiencing nomophobia, a high proportion are at risk of developing nomophobia [5, 7].

Unfortunately, though there has been a statistically significant increase in Smartphone usage in the emerging nation of Pakistan, there is a dearth of research examining the levels of nomophobia in this population. Nomophobia is associated with lower academic performance and achievement [10, 11, 12, 13], difficulties in attentional regulation [14], greater levels of depression [15], anxiety [13, 16, 17], sleep problems [17], loneliness [4, 16, 18] and lower levels of happiness [4, 19, 20]. Research has also demonstrated that adolescents exhibit greater mental health problems on days when they used their technological devices less frequently [21].

In terms of vulnerability to nomophobia, higher levels of nomophobia have been found to be related to various demographic variables, including: gender, level of education, age, and frequency of use in various studies in India [4, 5, 8, 9, 13, 22, 23, 24]; however, there is again a scarcity of research into nomophobia and its correlates in Pakistan. In particular, women are notably more likely to experience Nomophobia [4, 5, 13, 25, 26, 27] because they do not want to feel lonely in public places, are afraid of losing contact with others and do not want to give up their comfort [13, 26]. Also, those with higher levels of education are more likely to purchase and use Smartphones [1] and those within the age bracket of 18–34 have the highest rates of internet use [1]. Moreover, frequency of use has been shown to be a strong predictor of Nomophobia [9, 28]. Importantly, self-reported frequency of technology use has been found to be highly correlated with actual use [29] indicating that it is a reliable method of measuring Smartphone use as well as an important predictor of Nomophobia [28].

Due to the increase in usage of smartphones, especially among undergraduate students, and the negative impact that nomophobia has on multiple outcome variables in other populations, it is imperative to evaluate the level of nomophobia and its predictors in Pakistani undergraduate students. In elucidating levels of nomophobia and its predictors, psychologists and educators will be better equipped to understand this population. Importantly, possible therapeutic interventions and educational policies could be initiated at the primary, secondary and tertiary level of prevention.

## 2 Hypotheses

The current study aims to test the following hypotheses:

1. Women will use their Smartphones more frequently than men.
2. Women will be more likely than men to experience nomophobia.
3. Women and men will report differences within the four factors of nomophobia. This exploratory hypothesis posits to elucidate possible theoretical reasons for the gender differences seen rates of nomophobia.
4. Smartphone use (number of hours of usage and times per day checking) and length of time in university (credit hours) will predict nomophobia.

## 3 Materials and methods

### 3.1 Sample and procedure

Power analysis indicated that between 74 and 108 participants were required to compute a regression analysis with four predictors [30]. A sample of 156 undergraduate students participated in a larger study approved by the Forman Christian College (A Chartered University) Institutional Review Board (IRB reference number: IRB-72/04-2018) conducted between May 2018 and August 2019 on smartphones and cognition. The study protocol followed all ethical guidelines for human participant research, ensuring comprehension of informed consent, voluntary participation, confidentiality, and protection of data. There were no ethical violations over the duration of the study. Of note, the University is an English-medium, liberal arts university and therefore all questionnaires were in English and all majors of study were invited to participate. Participants were recruited via posters and information provided in Introduction to Psychology courses, as this course meets the general education requirements of the liberal arts education system and students from all major fields of study could therefore be invited to participate. Students who participated were offered extra credit that could be applied to courses within the Psychology Department.

Students participated by visiting the Cognitive Research Lab on the University campus and filling the required forms, including informed consent, demographics sheet (i.e., questions relating to age, gender, year of study, hours of usage per day, and number of times per day checking phone), and the Nomophobia Questionnaire [2, 3]. Several participants were excluded due to missing data (14) or extreme scores (4; Mahalanobis Distances of *p* < .001), resulting in a sample of 138 students. Descriptive statistics of the sample are displayed in Table 1.

**Table 1:** Descriptive statistics of the sample.

Variables	M	SD	Frequency (%)
Age	20.43	1.93	
Sex			
Male			45 (32.60)
Female			92 (66.70)
Semester			
Freshmen			70 (52.63)
Sophomores			14 (10.10)
Juniors			22 (15.90)
Seniors			27 (19.60)
Hours of phone usage per day	6.94	4.10	
Times per day checking phone	41.55	46.13	

One participant declined to report their gender, four did not complete the NMP-Q, and five participants did not report their year of study. The average age was 20.43 years (SD = 1.93), and 92 of the students were women (66.7%), and 42 were men (32.6%). A large proportion were Freshmen (70; 52.63%), followed by Seniors (27; 19.6%), Juniors (22; 15.90%), and Sophomores (14; 10.10%). On average students reported spending 6.94 (SD = 4.10) hours a day on their smartphones and checking their smartphones an average of 41.55 (SD = 46.13) times a day.

### 3.2 Measurement tool

#### 3.2.1 The Nomophobia Questionnaire (NMP-Q) [2, 3]

The NMP-Q [2, 3] is a 20-item self-report measure that can be used to assess the severity of nomophobia or fear of being without one's phone. It provides a quantitative score ranging from 20 - 140, with higher scores indicating greater severity. The NMP-Q has been shown to have internal consistency (*α* = .945) [2,3] and construct validity (r = .710) [2,3]. In the current study, the reliability was excellent (*α* = .907). The NMP-Q consists of four primary factors or dimensions: not being able to communicate, loss of connectedness, not being able to access information, and giving up convenience [2, 3] each of which showed good internal consistency in the present study (*α* = .835, .837, .780, and .760, respectively), similar to previous research findings (*α* = .939, .874, .827, and .814, respectively) [2, 3].

### 3.3 Data analysis

IBM SPSS Statistics Subscription was used to compute all statistical analyses. Independent samples t-tests were used to test the first, second, and third hypotheses. Multiple regression was utilized to test the fourth hypothesis. For all hypothesis tests, a standard two-tailed significance level of *α* = .05 was set.

## 4 Results

### 4.1 Participant characteristics

Descriptive statistics of the NMP-Q scores of the sample are presented in Table 2. All students reported some level of nomophobia, with the largest proportion (59.4%) reporting moderate levels (NMP-Q total = 60-99), followed by severe levels (26.1%; NMP-Q total >100) and mild levels (11.6%; NMP-Q total = 21-59). No students reported absence of nomophobia (NMP-Q total <20).

**Table 2:** Descriptive statistics of the NMP-Q.

Variables	M	SD	Frequency (%)	Reliability (α)
Nomophobia Level				
Absence of Nomophobia			0 (0.00)	
Mild			16 (11.60)	
Moderate			82 (59.40)	
Severe			36 (26.10)	
NMP-Q Total Score	84.98	22.03		.907

*Note.* NMP-Q = Nomophobia Questionnaire.

### 4.2 Gender and smartphone use

An independent samples t-test was computed to compare men and women on average times per day checking their Smartphone and number of hours spent on their Smartphone. There was no significant difference between men (M = 50.73, SD = 52.05) and women (M = 37.28, SD = 42.80) on number of times they checked their Smartphone in a day [t (119) = 1.415, p = .16]; number of hours spent checking also did not reach significance [t (135) = -1.837, p = .068] with women reporting an average of 7.41 h (SD = 4.13) and men an average of 6.06 h (SD = 3.92).

### 4.3 Gender and NMP-Q total scores

An independent samples t-test was computed to compare men and women on average NMP-Q score. There was a significant difference between men (M = 80.42, SD = 22.28) and women (M = 88.88, SD = 21.53) on level of nomophobia [t (135) = -2.135, p < .05, Cohen's d = 0.4], with women reporting higher levels of Nomophobia with a small to medium effect size.

### 4.4 Gender differences within the factors of the NMP-Q

Given the significant difference between men and women on overall NMP-Q scores, further analyses were computed to compare men and women on the four underlying factors of the NMP-Q. Independent samples t-tests to evaluate gender differences within the four factors of the NMP-Q (see Table 3) were significant only for Factor 1 (Not being able to communicate), with women (M = 30.50, SD = 7.192) experiencing higher levels of fear of not being able to communicate than men (M = 26.67, SD = 8.50), though the effect size was small (Cohen's d = .01). All other comparisons were not significant.

**Table 3:** Independent Samples t-tests Comparing Men and Women on the Four Factors of the NMP-Q.

NMP-Q Factor	Male		Female		t-test	Effect
	M	SD	M	SD		Size
Not being able to connect	26.67	8.50	30.50	7.19	-2.757∗	.01
Losing connectedness	16.84	7.35	17.71	7.53	-.634	
Not being able to access information	17.51	5.24	18.96	5.57	-1.454	
Giving up convenience	19.80	6.81	21.96	6.62	-1.773	

*Note:* Effect size was computed using Cohen's d. NMP-Q = Nomophobia Questionnaire.

∗ Significant at the p < .01 level.

### 4.5 Demographic variables as predictors of NMP-Q

Prior to computing a multiple regression to evaluate the extent of the demographic variables' prediction of NMP-Q scores, evaluation of assumptions was examined. As noted earlier, four outliers outside 3.5 standard deviations were removed (Mahalanobis’ Distances with *p* < .001). Assumptions of normality, homogeneity of variance, and linearity were met. Pearson Correlation Coefficients computed between NMP-Q, age, number of credit hours, hours of usage per day, and number of times per day checking phone indicated that all the variables except age (*r* = .083) had weak positive relationships with total NMP-Q total score (see Table 4). Specifically, number of credit hours was weakly associated with NMP-Q scores (*r* = .209), as was hours of usage per day (*r* = .295), and number of times per day checking phone (*r* = .198). There were no correlations of *r* = .9 or greater, indicating that none of the variables were multicollinear or singular.

**Table 4:** Pearson correlation coefficient matrix of study variables.

Variable	1	2	3	4
1. NMP-Q Total				
2. Age	.083			
3. Number of Credits	.209∗	.664∗∗		
4. Hours of phone usage per day	.295∗∗	.082	.307∗∗	
5. Times per day checking phone	.198∗	.140	.226∗	.358∗∗

*Note.* NMP-Q = Nomophobia Questionnaire.

∗ Significant at the .05 level.

∗∗ Significant at the .001 level.

A multiple regression was therefore computed to predict NMP-Q scores based on hours of usage per day, number of times per day checking phone, and number of credit hours (see Table 5). Age was excluded as it had no significant relationship with NMP-Q scores [31].The regression equation was significant [R = .331, R^2^ = .109, F (3, 116) = 4.748, p < .05]; however, hours of usage per day (ß = .226, p < .05) was the strongest and only significant predictor of NMP-Q. As number of hours increased, so did nomophobia scores.

**Table 5:** Multiple regression analysis of predictors of nomophobia.

Variable	Nomophobia			
	B	SE B	ß	t
(Intercept)	73.132	3.946		18.534
Hours of phone usage per day	1.213	.521	.226	2.329∗
Times per day checking phone	.043	.045	.091	.957
Number of credits	.058	.045	.119	1.283

*Note.* R = .331, R^2^ = .109, F (3, 116) = 4.748, p < .05.

∗ Significant at the .05 level.

## 5 Discussion and implications

In the current study, the entire sample reported some level of nomophobia, with the majority reporting moderate to severe levels of nomophobia. This important finding demonstrates the need for further monitoring of the psychological implications of smartphone ownership within other populations of Pakistan. This study also uncovers the need for increased awareness and research into the effects of smartphone use on psychological health, particularly in developing nations where there is a dearth of research and psychological awareness into this growing phenomenon. According to the World Health Organization [32] only 0.4% of health expenditures in Pakistan are allocated toward mental health. Education campaigns about the detrimental effects of Smartphones would not be possible at the governmental level with such limited allocation of resources, however, educational facilities such as universities and colleges could fill this need by creating awareness campaigns and implementing policies that encourage a reduction in Smartphone usage.

Interestingly, though no significant gender differences were seen in smartphone use (number of hours of usage, number of times checked), significant differences in nomophobia scores were seen between men and women. This is in line with previous research in other contexts which has found that women tend to report higher levels of nomophobia [4, 5, 25, 26, 27].

Importantly, the current study endeavored to further understand gender differences within the specific factors of nomophobia. Though women reported higher levels of overall nomophobia, they specifically demonstrated statistically higher scores on the subscale “Not being able to communicate” (Factor 1). This gender difference highlights a possible connection between phone use and relatedness among women and the importance these young women place in staying connected through their devices. Research suggests that many women in Pakistan are defined by their relationship with others, such as *wife*, *mother*, or *daughter* [33]. It is possible that the higher rates of anxiety women in this study had about “not being able to communicate” could be related to their relational position in society. Importantly, it is also possible that there might be severe consequences for lack of communication, due to the importance given to honor within this context and the severe consequences if there is even a hint of a breach of honor [34, 35]. Furthermore, high rates of violence against women have been recorded as 93% women report having experienced some form of sexual violence in public places in their life [36] and, 5508 cases of kidnappings of women from 2004 to 2016 were reported [37]. Therefore, it is also possible that mobile phones are a source of comfort and safety to women because it enables them to reach someone if they are in danger. A survey of 11 countries, including India, confirmed this as it found that 68%–94% women feel safer when they have a mobile phone as it could help them contact someone if there was an emergency [38].

The impact of nomophobia on young women in Pakistan is a particularly important finding given their relatively disadvantaged and marginalized position in society and the level of stress and pressure they already experience. As previously noted, rates of violence against women are high and the patriarchic social structure of Pakistan allows for this sanctioned violence and systemic marginalization of women [34]. Extreme examples of this can be seen in cases of “honor killings” in which women are killed for bringing shame on their families [35]. Given women's status within society and their significantly higher levels of nomophobia, it becomes even more important to target awareness and treatment interventions toward this disadvantaged group.

Finally, though this study found that significant relationships exist between number of credits, hours of usage per day, and number of times per day checking phone and NMP-Q scores, only hours of usage per day significantly predicted nomophobia. Previous research has uncovered similar results [9, 29], suggesting that, similar to other behavioral addictions, the amount of time spent on the addiction leads to increased anxiety during abstinence [2, 3, 18]. This suggests the importance of educating students about managing the amount of time they spend on their smartphones and has policy implications at the university level; however, it should be noted that these policies should not be draconian in nature, such as the complete ban in 2014 on teachers having cell phones in schools [39]. Appropriate policies would need to be decided at the university and faculty level and should take into account the autonomy and responsibility students have for their own well-being. It is especially important, given the rapid increase in Smartphone use in this nation, that education and awareness about the real impact Smartphones have, as well as methods of monitoring and reducing use, should be a priority.

As with other behavioral addictions, the importance of social pressure and the social context of Smartphones leading to connectedness, should be taken into consideration for treatment. In the case of Smartphones, which have many benefits as well as risks, abstinence would not be a practical strategy as parents and friends often expect students to be available whenever they call; however, a harm-reduction method, such as those use in adolescent substance use and other health behaviors, could be considered [40]. At the primary prevention level, this includes educating students and encouraging appropriate use. Strategies such as applications that monitor screen time, as well as parental monitoring of use would be important at this level. At the secondary and tertiary levels of prevention, the focus would be on educating students about the harms of excessive use and, ultimately, giving students strategies to reduce the number of hours students spend on their phones (i.e., screen time monitoring software, setting alarms, including family in the process).

## 6 Limitations and future directions

There are several limitations of the current study. Only students from a private university in Lahore were included in this study so generalizations cannot be made to other socio-economic groups or areas of the country. Future studies should consider recruiting a more diverse sample, including using questionnaires in Urdu or other regional languages, to aid in characterizing the scope and breadth of this rapidly emerging issue.

Importantly, given the high levels of nomophobia in this sample, it is necessary to consider other possible predictive factors in order to better understand the risks for nomophobia. This study has revealed moderate to severe levels of nomophobia, indicating a need to also consider the possible impact of these rates of nomophobia on aspects of students’ academic success, and social, emotional, and psychological health and well-being. Future studies should be conducted to further understand the impact of the growing use of Smartphones within this population.

## 7 Conclusions

The current study has shown high levels of nomophobia among Pakistani undergraduates, particularly among young women. The strongest predictor of nomophobia was number of hours spent using the Smartphone. This highlights the importance of education and raising awareness of nomophobia, its causes, and possible methods of reducing the negative impact of Smartphone use.

## Declarations

### Author contribution statement

E. Schwaiger: Conceived and designed the experiments; Analyzed and interpreted the data; Contributed reagents, materials, analysis tools or data; Wrote the paper.

R. Tahir: Performed the experiments; Wrote the paper.

### Funding statement

This research did not receive any specific grant from funding agencies in the public, commercial, or not-for-profit sectors.

### Competing interest statement

The authors declare no conflict of interest.

### Additional information

No additional information is available for this paper.
